# Supplementary material for: Acceptance and compliance with micronutrient powder (MNP) among children aged 6–23 months in northern Nigeria
Source: PLOS Glob Public Health. 2022 Oct 17;2(10):e0000961. doi: 10.1371/journal.pgph.0000961 (PMC10022258; doi:10.1371/journal.pgph.0000961)
Supplement: S1 Dataset — (ZIP) [file pgph.0000961.s008.zip › S8_Dataset/Direct Observations.zip!/Direct Observations/280615_K_DW_SA_1_DO.pdf]

1.1.1 Instrument F –Direct Observation Form

**<<Instrument F Direct Observation Form>>**
**PI Name:** PragyaMathema,Stephen Kodish<br>**Study Title:** Formative research to develop an infant and young child feeding (IYCF) intervention with micronutrient powder (MNP) for children aged 6 – 23 months<br>**PI Version/Date:** Version1.0/January 24, 2015

Date: 28/06/15 Location: Biki

Data Collector: Samira Adamu

Main goals of this direct observation are to answer the following questions:<br><br>1. In <u>this</u> household, what would be the best way(s) to give nutritional supplements to <u>young children</u>? Why?<br><br>2. In <u>this</u> household, describe the food sharing that you observe throughout the meal. Are there any <u>predictable</u> patterns?<br><br>3. In <u>this</u> household, who is favored and how is this favoritism expressed during meal preparation and eating?

Household Composition Table (Fill out <u>as</u> you do data collection)

INITIALS	SEX (M/F)	AGE (estimated)	Relationship to household	Description (not necessary)
BB	M	22months	Youngest child in the house	Wearing jersey shirt and blue shot
MB	F	29 years	Mother of the (2nd wife) child	Wearing green and red attire
W1	F	30years	First wife of the house	Wearing light green and orange wrapper
W2	F	15years	Youngest wife	Wearing blue and white attire
DL	F	16years	Wife of the eldest son	Wearing green top and maroon trouser
BB2	M	6years	Son of the house	Wearing light blue shirt and trouser
BB3	M	6years	Son of the house	Wearing dark purple and black attire
BB1	M	7years	Son of the house	Wearing black shorts

BB4	M	17years	Youngest child (step brother)	Wearing green and milk shorts
FB	M	50years	Father of the house	
BG2	F	4years	Daughter of the house	
BG1	F	7years	Daughter of the house	Wearing black and white hijab
BG3				

PARTICIPANT ID: _________

TIME	ACTOR INITIALS	RECIPIENT INITIALS	WHAT HAPPENED	FOOD	Reactivity
8:40	MB		Carrying the child on her laps and sieving grounded millet flour to remove coarseness		
	W1		Arranging locally made clay pans, and bowls into local oven made of clay and sand with ignited charcoal in the oven to bake pans, pots and bowls.		
	W2		Cutting black selotape into different sizes which is used in making henna decorations for women on the leg		
	DL		Pounding some of the coarse flour obtained after i.e being sieved by the step mother in-law		
	BB3	BB4	Feeding the child with kunun millet	Kunun millet	
8:45	BB		Playing with locally made wheel fixed with stick using a nail and eating a fruit called 'kade" around the house	'kade'	

TIME	ACTOR INITIALS	RECIPIENT INITIALS	WHAT HAPPENED	FOOD	Reactivity
			while running and directing it just like a ride		
	BB3	BB4	Feeding the child with kunun millet	Kunun millet	
	W1		Sweeping around the locally made oven		
	W2		Still cutting the selotape for patterns use for henna decoration for women on her leg (foot)		
	MB		Still sieving the grounded millet flour		
	DL		Still pounding the coarse millet flour to extract more fine flour after being sieved again		
	BB1&BB2		Playing ball together in another corner of the house		
8:50	BB		Crying because he fell on the floor while riding his locally made wheel pusher		
	MB		Arranging locally made clay pan, bowls and pots into another locally made oven to bake the clayed pans		
	DL		Sieving the pounded coarse flour to extract more flour		
	W2		Going towards her apartment with a friend/guest		
8:55	BB		Playing in the daughter in-law's apartment with his locally made wheel pusher and eating 'kade'	Shea butter food	
	DL		Spreading clothes on the rope tied to different walls for drying clothes and liners		
9:00	W2		Putting a small cup full of the properly mixed maize mixture which is semi solid into each of the small pans and bowls arranged into the locally		

TIME	ACTOR INITIALS	RECIPIENT INITIALS	WHAT HAPPENED	FOOD	Reactivity
			made oven and allowed to bake by covering with tray		
	MB		Also mixing another maize paste with salt and kanwa(potash)		
	BB		Pushing the wheel and crying while moving towards the mother, because she is calling him to sit beside her as she mixing the maize paste		
	BB1,BB3&BB2		Climbing the 'kade' fruit tree and dancing to a song being played from outside the house		
9:05	DL		Pounding the small remaining coarse millet flour		
	W1	BB4	Sweeping her room while backing the child		
	BB1,BB2&BB3		Still dancing on the 'kade' fruit tree while plugging the kade fruit and dropping them on the floor		
	BB		Playing with sticks standing close to the mother while she is covering the oven with tray and placing a blanket over the tray		
	DL		Backing the baby from the neighborhood and sieving the immediate pounded coarse millet flour to extract more fine flour		
	W1		Still sweeping the front of her room		
	BB2&BB3		Sitting on the kade tree and eating plugged kade fruit and gisting with the sisters down	Kade	
9:10	BB		Sitting on the floor and playing with sieve and sand		
	BB2&BB3		Still sitting on the branches and eating kade fruit	Kade fruit	

TIME	ACTOR INITIALS	RECIPIENT INITIALS	WHAT HAPPENED	FOOD	Reactivity
	MB		Sweeping the compound of the house		
	W1		Sitting on a stool in front of her house		
9:15	BB		Sitting on the floor and drinking kunu alone		
	MB		Still sweeping the compound floor		
	W1		Sitting in front of her room		
	BG1&BG2		Plugging the kade fruit using a long stick		
	DL		Making a paste of the millet flour grounded and sieved by adding water and mixing it properly with water to obtain a semi solid paste which is a bit thick		
9:20	BB	BB	Still drinking kunu alone	Kunun millet	
	MB		Washing the pots and bowls used for mixing of maize mixture which is called 'hochi'		
	DL		Pouring the millet paste in a small bowl under the sun		
	BB4		Playing with shaft of millet in a bowl		
9:25	BB		Drinking kunu and playing with stick		
	W1		Removing the baked hochi from the locally made oven and arranging on a tray		
	F		Entering the house with hoe on his shoulder carrying animal feed towards the animal farm		
9:30	BB		Sitting close to the father and taking kunu along side 2 other siblings talking with the dad		
	W1		Using spoon to remove the		

TIME	ACTOR INITIALS	RECIPIENT INITIALS	WHAT HAPPENED	FOOD	Reactivity
			baked hochi from the pans		
	DL		Sitting and watching the step mother in-law		
	W2		Gisting with the husband and playing with the child		
	MB		Sweeping a corner of the compound under the tree		
	BB		Playing with dad's slippers on the floor		
9:35	BB1		Playing and eating kade fruit in front of the house		
	FB	BG1,BG2&BB4	Playing with the children		
	DL		Making beads necklace		
	MB		Removing baked hochi from the locally made oven and penalty arranging it on a mat		
	W2	BB4	Wearing pant for the child		
9:40	BB		Sitting near the daughter in-law and eating kade	Kade	
	MB		Using spoon to remove the 'hochi' from the clay pans		
	W1	BB1	Beating him with a stick and making him wash his hand with water and sand after the using the toilet		
	W2		Mixing the henna (lalle powder) with water		
	FB		Sitting and watching the activities of the house		
9:45	BB	DL	Lying on her lap and eating kade fruit	Kade	
	BB3	BB3	Eating tuwo and miyan kadi	Tuwo and miyan kadi	
	W1		Arranging the hochi in a transparent polythene bag		
	MB				

TIME	ACTOR INITIALS	RECIPIENT INITIALS	WHAT HAPPENED	FOOD	Reactivity
	FB		Sitting on a plastic chair and watching the activities of the house		
9:50	BB		Sitting near brother while he eats, playing with rope		
	W1	BB3	Sitting on a stool in front of her room and talking to the boy to finish eating and go hawking hochi		
	FB		Sitting on a plastic chair and watching then household		
	MB		Draining the pots and big sieves she washed		
	DL		Making necklace with beads and thin rope		
9:55	BG1		Eating tuwo and miyan kadi sitting on a stool	Tuwon maize and miyan kadi	
	W1		Sitting on a stool in front of her house		
	FB		Sitting on a plastic chair and watching the activities in the house		
10:00	BB1		Standing near the father and playing with handsets		
	BG1		Still eating tuwo and miyan kadi		
	MB		Arranging the hochi in a big transparent polythene bag		
	BB2		Packing fruit from the floor and putting them in a bowl		
	BB1		Playing with a used rubber bowl of soup		
10:05	BB		Standing and playing with handset and started stooling		
	MB		The mother is removing the pant and sitting him on a plastic stool bowl while she		

TIME	ACTOR INITIALS	RECIPIENT INITIALS	WHAT HAPPENED	FOOD	Reactivity
			scolds the child		
	W1		Counting the hochi that would take for hawking		
	DL		Still making bead for necklace		
	W2		Sitting in front of her room and fixing lalle		
10:10	BB		Sitting on a plastic stooling pan and drinking kunun millet in a cup	Kunun millet	
	MB		Using spoon to remove the hochi from the clay pans and bowls		
	BB1		Sitting close to the father and playing with hand set		
	BB3		Carrying the basin of hochi and crying because he doesn't want to go		
	DL		Making bead for necklace		
	W2		Sitting in front of her room and fixing lalle		
10:15	MB	BB	Carrying a kettle of water and the plastic stooling bowl to one corner of the house while the baby follows her to wash and clean him up after stooling		
	DL&W1		Buying/pricing wrappers, shoes, veils etc from a hawker who came to the house to sell		
	W2		Still sitting in front of her room and fixing lalle		
	FB		Sitting on a plastic chair and talking to the children		
	BB2&BG2		Playing and beating each other together		
	BB4		Crying because he was not allowed to play		
10:20	BB2&BG2		Beating each other while the father scolds		

TIME	ACTOR INITIALS	RECIPIENT INITIALS	WHAT HAPPENED	FOOD	Reactivity
	BB		Sitting and playing with locally mad hand fan		
	MB		Still removing the hochi from clay pans using spoon		
	W1&DL	BB4	Sitting and looking at the hawkers clothes and veils while she breast feed her child	Breast milk	
10:25	BB		Playing with step mother's handset		
	W1&DL		Sitting and still checking the clothes and shoes while she breast feed her baby	Breast milk	
	MB	BG1	Carrying a basin of hochi and placing it on her head for hawking		
	FB		Sitting on a plastic chair and watches everyone in the house		
10:30	BB		Crying and following the mother as she packs her bowls and plates from the basket where rained them in her apartment Carrying a basin of hochi and placing it on her head for hawking		
	BB1	BB4	Playing with empty carton of sphagetti		
10:35	BB		Still crying and following the mother as she enters her room		
	BB		Playing with an empty bottle of yoghurt in the room		
	MB		Arranges her dishes, bowls and plates on the cabinet		
10:40	MB		Still arranging her things on the cabinet		
	BB		Playing with her brush on a cane bed and crying		
	FB		Entering the room and asking the mother for his cap		
10:45	MB	BB	Sitting on a mat in front of her	Kunun	

			room and feeding the child kunu in a cup mixed with a sachet of MNP<br>The kunu is cold and its semi solid, the child doesn't want to take but she forced her to finish it	millet	
	BG2		Eating tuwon maize and miyan kadi		
10:50	MB	BB	Still forcing the child to finish the kunu.		
	BG2		Asking the mother of more tuwo but she gave the remaining kunu with MNP while saying finish the tsakin kunu since the child doesn't take the tsaki		
10:55	MB		Sweeping her room again		
	BB		Playing with the cane bed		
11:00	MB		Sweeping the frontage of her room		
	BB		Playing with stick and hair brush on the cane bed		
11:05	BB		Crying on falling down from the bed		
	MB	BB	The mother raising him and wiping his tears		
11:10	MB		Still sweeping the compound of her apartment		
	BB	BB	Sitting on a plastic chair in front of the child mother's room and carrying the child on his laps while he plays with handset		
11:15	FB	BB	Sitting on a plastic chair in front of the child's mother's room and carrying the child on her laps		
		BG2	Sitting on a small stool		
		MB	Carrying a basin of water		
11:20	MB	BB	Bathing the child while the child is crying and the sister is telling him to stop crying		

11:25	MB	BB	Backing the child and removing the daughters pant and vest		
11:30	MB	BB	Backing the child and bathing the daughter		
11:35	MB	BB	Still backing the child and bathing then baby		
	FB		Sitting and listening to radio		
11:40	MB	BG2	Wearing clothes for her while she is still backing the child and sitting on a cane bed		
11:45	MB	BB	Backing the child and washing the children's shoes		
11:50	MB	BB1,BG2	Sitting on a stool carrying the child on her laps and rubbing Vaseline for the daughter in front of her room		
11:55	MB	BB	Sitting on a stool and rubbing Vaseline on his body on her laps		
12:00	MB	BB	Cutting his finger nails while he sleeps on her laps		
12:05	MB	BB	Cutting his toe nails while he sleeps on her laps		
12:10	MB	BB	Sitting on a stool and carrying the child on her laps while he sleeps		
12:15	MB	BB	Sitting on a stool and carrying the child on her laps while he sleeps		
12:20	MB	BB	Sitting on a stool and carrying the child on her laps while he sleeps		
12:25	MB	BB	Still Sitting on a stool and carrying the child on her laps while he sleeps		
12:30	MB	BB	Still Sitting on a stool and carrying the child on her laps while he sleeps and gisting with the daughter inlaw in front of her room in her apartment		
12:35	MB	BB	Still carrying the child on her laps in her apartment		
12:40	MB	BB	Still carrying the child on her laps in her apartment		
12:45	MB	BB	Still carrying the child on her laps in her apartment		

12:50	MB	BB	Still carrying the child on her laps in her apartment		
12:55	MB	BB	The child is still sleeping on his mother's leg in her apartment		
1:00	MB	BB	The child is still sleeping on his mother's leg in her apartment		
1:05	MB	BB	The child is still sleeping on his mother's leg in her apartment		
1:10	MB	BB	The child is still sleeping on his mother's leg in her apartment		
1:15	MB	BB	The child is still sleeping on his mother's leg in her apartment		
1:20	MB	BB	The child is still sleeping on his mother's leg in her apartment		
1:25	MB	BB	The child is still sleeping on his mother's leg in her apartment		
1:30	MB	BB	Still carrying the child on her laps while he sleeps and selling shea butter to a girl from the neighborhood		
	BB1,BB2&BG2		I could see the children on the 'kade' fruit tree from the mother's apartment while sitting on a stool		
	MB	BB	Still carrying the child on her laps while he sleeps		
	BG4		Standing beside the mother and crying		
	BB4		Coming into the apartment with a cup		
1:35	MB	BB	Still carrying the child on her laps while he sleeps		
	BG2		Still standing beside the mother and dragging her clothes		
1:40	MB	BB	He is still sleeping on his mother's laps		
1:45	BB		Crying on a stool as he just woke up		
	DL		Coming to apartment, carried the child and is giving him water to drink	Water	
	MB		Also coming into the apartment with a bowl of kunu and the		

			daughter is following her about crying		
1:50	BB	BB	Feeding herself kunun sorghum	Kunun dawa	
1:55	BB	BB	Feeding herself kunun sorghum in the compound of the house outside the apartment		
2:00	BB	BB	Drinking kunu and crying		
	MB		Carried the child on her laps and started feeding the child with kunu	Kunun dawa	
2:05	BB		Crying and following the mother around the house		
	MB		She is ccarrying a kettle of water for the husband's ablution		
	W1		Fetching water from the well		
	BB4	BB	Playing with stick and hitting the child		
	DL		Sweeping another corner of the house where the children ate kade fruit		
	BB		Still crying and shouting at the mother for food		
	BB4		Touching my bag and my veil		Because he would not have done that if I was not around
	BG2,BG3		Drinking kunu together from the same bowl		
	W2		Performing ablution under the tree		
	MB		Cutting tuwon masara into small balls and adding fried shea butter oil and salt		
2:10	BB	BB	Eating tuwon masara and shea butter oil with salt		
	BB4	BB4	Drinking kunun sorghum on the floor		
	BG2&BG3		Eating kade fruit		

	DL		Mixing freshly prepared shea butter oil for cooking		
	W1		Sitting on a stool and also mixing freshly prepared shea butter which was prepared yesterday from the shea butter nut after it has been grinded in a grinding machine and made into a semi solid paste and put in a pot on fire where it is allowed to cook until fractionation occurs and the oil is extracted		
2:15	W1	BB4	Breastfeeding the child	Breast milk	
	BB	BB	Eating tuwon masara and shea butter oil mixed with salt		
	DL		Mixing the processed shea butter used for cooking		
2:20	BB	BB	Eating tuwon masara and shea butter oil mixed with salt		
	W1		Mixing another portion of the processed shea butter		
2:25	BB	BB	Still eating tuwon masara and shea butter oil mixed with salt		
	W1		Cutting tuwo masara into small balls and breast feeding her baby because she is crying	Breast milk	
2:30	W1		Mixing the balls of tuwon masara with shea butter and salt		
	BB		Drinking water from the water kettle		
	MB		Buying palm oil, pepper and magi from a girl who hawks them		
	BG2&BG3		Still playing with sticks and nuts of the kade fruit		
2:35	BB		Standing and crying and calling on his mother who was in the bathroom		
	W1	BB4, BG3&BG2	Giving the children tuwon masara and shea butter oil which she referred to as ‘tantu’	Tantu	
	BB1		Eating ‘tantu’ seperately	Tantu	

2:40	BB		Still crying and shouting as mother gave him a smack with her hand and continued feeding him		
	BB4, BG3&BG2		Still eating ‘tantu’ together		
	W1		Carrying a basin with sieve and sack of grain flour		
2:45	MB	BB	Still feeding the child tantu although he is still crying		
	W1		Sieving the maize flour		
	BB4,BG3,BG2		Still eating tantu together		
2:50	MB	BB	Giving the child water from a rubber kettle	Water	
	W1	BB4	Sitting on a stool and breastfeeding her child while sieving the maize flour	Breast milk	
3:05	BB		Still crying and calling mother’s name		
	MB		Sitting beside the child and giving him water	water	
3:10	W1	BB4	Still breastfeeding and sieving the maize flour	Breast milk	
	BG3		Lying down on the pavement and playing		
	BG2		Drinking water		
	BB		Still crying and eating ‘tantu’ again		
3:15	BB		Playing with the food and throwing some on the floor		
	W1		Backing the child and sieving maize flour		
	BG2&BG3		Playing together		
3:20	MB		Sitting beside the child as he plays with stick and mother’s wrapper		
	W1	BB4	Backing the child and sprinkling water on the sieved maize flour		
	BG2&BG3		Playing with sand and small clay pot		

3:25	BB		Playing with sand and the remaining tuwon masara		
	W1	BB4	Backing her child and mixing the maize flour with water to form a thick paste		
	MB		Stirring semi solid millet paste kept under the sun by the daughter in-law in the morning		
	BG2&BG3		Playing with sand and small clay pots		
3:30	BB		Crying and calling mother's name		
	MB		Lying on a mat in another corner under the shade		
	W1	BB4	Lying on the mat with her child		
3:40	BB		Lying down near the daughter in law on the mat and eating cooked tafasa	tafasa	
	MB		Lying down on the mat		
	W1	BB4	Lying on the mat with her child		
	BG2		Practicing 'henna' decoration on her hands		
3:45	DL	BB	Happily playing with the child		
	MB		Lying on the mat and taking a nap		
	W1	BB4	Lying on the mat and taking a nap		
3:50	BB		Lying near the daughter in-law and playing with her		
	MB		Sleeping on a mat		
	W1	BB4	Sleeping on a mat with her child		
	BG2&BG3		Mixing water and sand to form a ball		
3:55	DL	BB	Carrying the child to then entrance of the house		
	MB		Still sleeping on the mat		

	W1	BB4	Still sleeping on the mat		
4:00	DL	BB	Still playing with the child		
	MB		Still sleeping on the mat		
	W1	BB4	Still sleeping on the mat		
4:05	DL	BB	Still playing with the child		
4:10	DL	BB	Still playing with the child		
	W1	BB4	Still sleeping on the mat		
4:15	BB		Crying and beating the mother to wake up		
	MB		Lying him closer		
	W1	Bb4	Still sleeping on the mat		
4:20	BB		Lying with the child on the mat		
	W1	BB4	Still sleeping on the mat		
4:25	BB		Lying with mother on the mat and crying while the mother is still dozing off		
4:30	BB		Lying with the mother on the mat and hitting her with a small stick to wake up		
	W1		Carrying a kettle of water to the bathroom		
	W2		Sitting on a stool		
4:35	BB		Playing with dry battery cell while the mother is still lying on the mat		
	W2		Sweeping the frontage of her apartment		
4:40	FB		Entering the house with a bowl of waina	waina	
	MB		Welcoming him and collecting the bowl		

	BB		Crying and raising hands for the father to carry him		
4:45	BB	BB	Eating waina by himself	waina	
	FB		Sitting on a plastic chair under a shade		
4:50	BB		Sitting on the floor and eating waina and drinking kunu	Waina and kunu	
4:55	BB		Sitting on the floor and eating waina and drinking kunu	Waina and kunu	
	MB		Sitting beside the child as he eats		
	W1		Performing ablution		
5:00	BB		Sitting on the floor and eating waina and also playing with sticks	waina	
	MB		Sitting on the mat while the daughter is crying and asking for tuwo even without tantu		
5:05	BG2		Carrying a pot of remaining miyan kadi soup and eating with tuwo		
	BB		Wearing his slippers		
	MB		Fetching water from the well		
5:10	BB		Sitting on his father's feet and playing with sand		
	W1&DL		Sitting on the same mat		
5:15	BB	BB2	Playing with brother who just entered the house		
	MB		Sweeping the cooking area to set fire		
5:20	BB1	BB	Putting the child into a mortar used for pounding and the child smiles happily		
	MB		Carrying firewood to the kitchen to set fire		
	W1	BB4	Breast feeding her baby	Breast milk	
5:25	BB		Playing in the mortar		

	MB		Setting the fire		
	W1	BB4	sitting on the floor and feeding the child	Breast milk	
	DL&W2		Sitting on the mat and gisitng		
5:30	MB		Adding more firewood to the ignited fire		
	BB		Moving around and pushing an empty bucket like a car		
	W1	BB4	Still breast feeding her child	Breast milk	
5:35	MB		Pouring water into the pot placed on fire		
	BB		Still playing around		
	W1,DL&W2		Sitting on a mat and gisting with the husband/father in-law		
5:40	MB		Adding more water to the pot placed on fire		
	BB	BB4	Playing with baby		
	W1,DL&W2		Still lying on a mat and gisting		
5:45	MB		Water is boiling on fire, making the millet paste prepared during the day more watery by adding small water and stirring with hand for it to mix properly		
	BB		Eating cooked cow skin with other children		
	W1,DL&W2		Still lying on the mat and gisting		
5:50	MB		Washing maize with water		
	BB		Standing at the entrance of the house with the father and 3 other siblings		
	MB		Washing maize with water and draining in a sieve		
5:55			Pot of water is still boiling		

	BB		Standing in the house and doing nothing		
6:00	MB, W1,DL&W2		All sitting on the mat and gisting together		

Primary Questions (Complete after you complete data collection):

From what you have seen observing THIS HOUSEHOLD, please answer the following questions in detail:

Question	Write your detailed response here
1. In this household, what would be the best way(s) to give nutritional supplements such as an MNP to young children to ensure compliance?<br><br>Why?	It should be through the mother and father<br><br>Because the mother is always with the child and she will make sure it is administered because during my stay for observation, she had mentioned improvement in the child's health since the therapy started; also the father also commented that he is in full support of the program. In fact, he asked me why only a child was given when there are two children of the required age group.<br>So I had to explain the program will soon be scaled up and all children will have access to the MNP because I realized he loves the children very much. During my stay he really supported me and was checking on me, that is why I feel he also can be used.
2. In this household, who is favored during meal preparation or feeding?<br><br>How is this favoritism expressed?	All the children are favored in the household although, the two younger ones are mostly favored.<br><br>If the child is eating, the mother always sits beside him or carries him on her laps or feeds him herself. Also, if anything is brought to the house, the two younger ones are fed before other children.

3. How could we promote the MNP supplement for this family that would attract their attention?<br><br>Who would they listen to?	The product should be given alongside incentives like bowls and spoons, or other eatables to improve their interest towards the product, although this household has high interest in the product. Also both caregivers should be given at the same time<br><br>The caregivers would listen to their husbands. For the husband, he has good level of understanding so I don't think he needs to be linked to someone before listening.
4. In this household, what would be the best ways to promote the MNP but limit how much it would be shared?<br><br>Why?	Proper sensitization and awareness on the usage and importance and effects should be clearly stated to the caregivers<br><br>Because if they are properly sensitized and apprehend a god knowledge and understanding of the product, the sharing of the product will surely be limited. The family are really interested in the product and am sure they will abide by the rule of no sharing.
5. What other issues and concerns did you see with this family that are important for us to consider with regard to introduction of an MNP?<br><br>Why?	Sharing is a major concern, although not directly sharing the MNP but the food. The mother gave the child kunu mixed with MNP and later gave her daughter the remaining one; assaying that there are small grains of the kunu based millet which the child doesn't finish all the time and that was why she gave the daughter.<br><br>The temperature and consistency of kunu should be well described, infact it should be shown practically and let the care care givers feel the required temperature because they don't consider that as being important during preparation.

6. Based on your experience observing this child today, do you believe reactivity negatively impacted your ability to see the food-related behaviors of the child?<br><br>Please explain your answer in detail.	No, there was no impact of negative reactivity towards food related behaviors<br><br>Because the child is allowed to eat without any obstruction by himself because he prefers eating on his own since he is still practicing to eat alone.
